# Impact of nonpharmaceutical interventions on inflammatory diseases of the nose

**DOI:** 10.1080/07853890.2025.2557517

**Published:** 2025-12-17

**Authors:** Lili Dai, Shuhan Liu, Liwei Chen, Xiaofeng Fan, Sirui Wang, Yawen Ding, Xiangwei Yi, Yongran Cheng

**Affiliations:** ^a^ Clinical Medicine Department, Hangzhou Normal University, Hangzhou, People’s Republic of China; ^b^ Department of Otolaryngology, Langxi County People’s Hospital, Xuancheng, People’s Republic of China; ^c^ School of Public Health, Hangzhou Medical College, Hangzhou, People’s Republic of China; ^d^ Department of Otolaryngology, Affiliated Hospital of Hangzhou Normal University, Hangzhou, People’s Republic of China

**Keywords:** COVID-19, masks, otolaryngology, pandemics, rhinitis, rhinosinusitis

## Abstract

**Aim:**

This study aimed to investigate the impact of nonpharmaceutical interventions (NPIs), implemented by the Chinese government during 2020–2022 to reduce the spread of coronavirus disease 2019, on the incidence of inflammatory nasal diseases, including chronic nonallergic rhinitis, allergic rhinitis, acute rhinitis, chronic sinusitis and acute sinusitis.

**Methods:**

Medical records from the Department of Otorhinolaryngology of the Hangzhou Normal University Affiliated Hospital were analysed to compare the incidence of the five nasal diseases between the pre-pandemic (January 2018–December 2019) and the pandemic prevention periods (January 2020–December 2022). The Chi-square test was used to compare incidence rates and sex/age distributions.

**Results:**

The incidence of chronic nonallergic rhinitis, chronic sinusitis and acute sinusitis decreased significantly during the pandemic prevention period (*p* < .001), while the incidence of allergic rhinitis and acute rhinitis increased significantly (*p* < .001). NPIs were protective factors for chronic nonallergic rhinitis, chronic rhinosinusitis and acute rhinosinusitis (relative risk (RR) < 1) but risk factors for allergic rhinitis and acute rhinitis (RR > 1). The proportion of male patients increased significantly for chronic nonallergic rhinitis and chronic sinusitis (*p* < .001). The age of the patients showed significant differences in the four diseases – chronic nonallergic rhinitis, allergic rhinitis, chronic sinusitis and acute sinusitis (*p* < .001).

**Conclusions:**

NPIs had a significant impact on the incidence and epidemiology of nasal inflammatory diseases, with significant differences in incidence rate, sex and age distribution between the pre-pandemic and pandemic prevention periods.

## Introduction

Coronavirus disease 2019 (COVID-19), a highly infectious disease, spreads rapidly via respiratory droplets and direct contact [1]. China has implemented various measures involving the entire society to cut off the virus’s transmission route to prevent it from spreading. These measures include almost everyone wearing masks in public places and prohibiting all forms of gatherings, including work, visitation and school activities [2]. Measures such as reducing population gatherings and implementing isolation and prevention have continued to be in effect since the Chinese government launched the ‘Dynamic Clearing’ plan on 11 December 2021 [3].

We have noticed that these long-term nonpharmaceutical interventions (NPIs) have had a certain impact on nasal health [4,5]. Wearing masks is the most important NPI. Hence, it has become a way of life for people worldwide since the beginning of 2020, resulting in a massive influx of masks on the market. The World Health Organization recommends using three main types of masks, such as cotton, surgical and N95 masks, all of which can effectively reduce the spread of COVID-19 via respiratory droplets [6]. Several studies have explored the possible physiological and psychological changes caused by wearing masks. As ear, nose and throat practitioners, we have noticed different quality of masks available on the market. The quality of some of the masks is such that even after covering the mouth and nose, people may inhale fabric dust from the mask, harming the health of the nasal mucosa [4]. Further, masks can change the physical environment around the nose [7], affecting the comfort of the nasal mucosa. Attention to hand hygiene, isolation at home, and reducing population gatherings can reduce some external stimuli to the nasal mucosa, such as exposure to allergens. Studies demonstrated that in 2022 the symptoms and incidence of allergic rhinitis might have decreased due to staying indoors and wearing masks [8]. At the same time, these NPIs also reduced the transmission of some respiratory pathogens between individuals [9].

The study aimed to retrospectively analyse the characteristics of patients who sought medical treatment, as well as changes in the incidence and characteristics of diagnosed nasal inflammatory diseases (with a focus on chronic nonallergic rhinitis, allergic rhinitis, acute rhinitis, chronic sinusitis and acute sinusitis) under the comprehensive impact of NPIs during the pandemic prevention period. This will allow us to objectively analyse the impact of NPIs on nasal health after the COVID-19 outbreak.

## Materials and methods

### Study participants and sample collection

The Hangzhou Normal University Affiliated Hospital is a comprehensive hospital, rather than a specialized hospital, for infectious diseases. During the COVID-19 outbreak, the patients visiting the Department of Otorhinolaryngology were non-COVID-19-infected individuals. This study collected relevant data from outpatients from the Department of Otorhinolaryngology of the Hangzhou Normal University Affiliated Hospital from January 2018 to December 2022.

*Data source*: Information of eligible patients was extracted from the hospital’s electronic medical record system, including the total number, gender, age and disease type. For patients with repeated visits, only the data at the time of the first diagnosis were included for statistical analysis. The period from January 2018 to December 2019 was designated as the pre-pandemic period, and the period from January 2020 to December 2022 was designated as the pandemic prevention period.

*Inclusion criteria*: Patients clearly diagnosed with chronic non-allergic rhinitis (symptoms such as nasal congestion, runny nose, sneezing and/or postnasal drip persist for more than 12 weeks without evidence of allergic rhinitis), allergic rhinitis (typical symptoms: nasal congestion, nasal itching, sneezing, runny nose with clear discharge, combined with skin prick test or serum specific IgE testing), acute rhinitis (typical symptoms of acute rhinitis (nasal congestion, runny nose, sneezing, low-grade fever, etc.), with symptoms typically lasting no longer than 10 days, excluding allergic rhinitis and sinusitis), chronic sinusitis (nasal congestion, purulent nasal discharge, facial pain or pressure, and decreased sense of smell, with symptoms lasting for more than 12 weeks, combined with CT scan results) and acute sinusitis (nasal congestion, purulent nasal discharge, facial pain or pressure, and loss of smell, with symptoms lasting for more than 10 days to 4 weeks, combined with CT scan results); The materials are complete.

*Exclusion criteria*: Patients with other severe systemic diseases (such as severe cardiovascular diseases, advanced malignant tumours, etc.) that may affect the manifestation or treatment of nasal diseases; patients with mental disorders who are unable to complete the diagnosis and treatment process or provide accurate medical history information; patients with nasal symptoms caused by non-inflammatory factors such as trauma.

### Data analysis

The outpatient data of chronic nonallergic rhinitis, allergic rhinitis, acute rhinitis, chronic sinusitis and acute sinusitis confirmed by diagnosis during the two aforementioned periods were screened and compared. The changes in the incidence rate of the five nasal diseases were compared between the two periods. Additionally, a horizontal analysis was conducted to examine the incidence characteristics of each disease. Changes in the sex and age of the patients for the aforementioned five nasal diseases during the two periods were compared. NPIs were considered the exposure factors for outpatients during the pandemic prevention period to analyse the influence of NPIs on nasal diseases.

### Statistical methods

The Chi-square test was employed to compare the incidence of five types of nasal inflammatory diseases between pre-pandemic and pandemic prevention periods. Relative risk (RR) and 95% confidence interval (CI) were used to analyse whether NPIs were protective or risk factors for each nasal inflammatory disease. A significance level of *α* = 0.05 was used, with *p* < .05 indicating a statistically significant difference. The data were analysed using IBM SPSS Statistics 26.0 (IBM Corporation, Armonk, NY), and the chart production was conducted using GraphPad Prism 9 software (GraphPad Software, La Jolla, CA).

### Ethics statement

This study was approved by the Ethics Committee and Information Department of the Hangzhou Normal University Affiliated Hospital. It was unnecessary to obtain signed informed consent from the patients, as this study only involved the number of outpatients rather than specific cases or patients.

## Results

**Figure 1.**
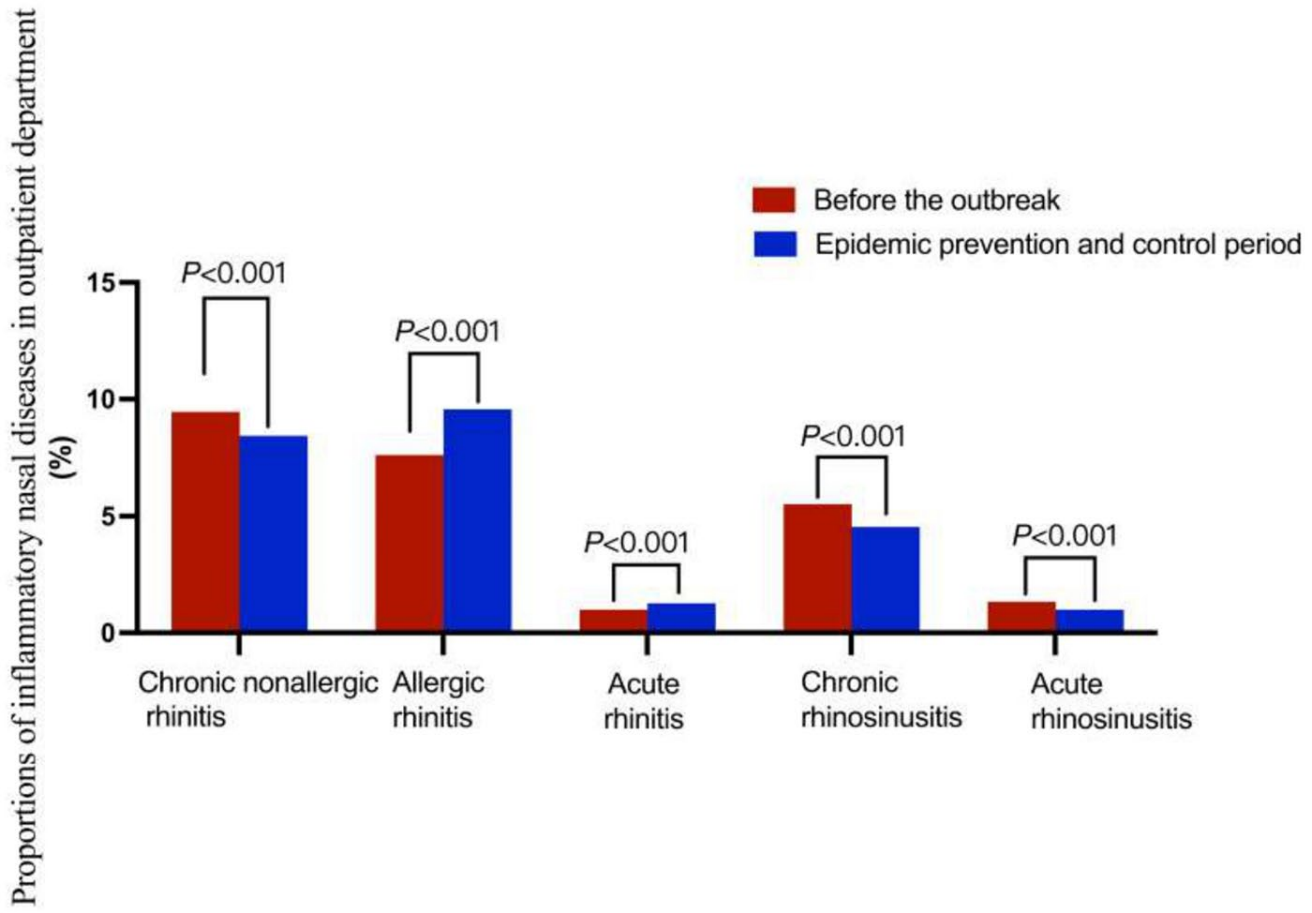
Proportions of inflammatory nasal diseases in the outpatient department.

**Figure 2.**
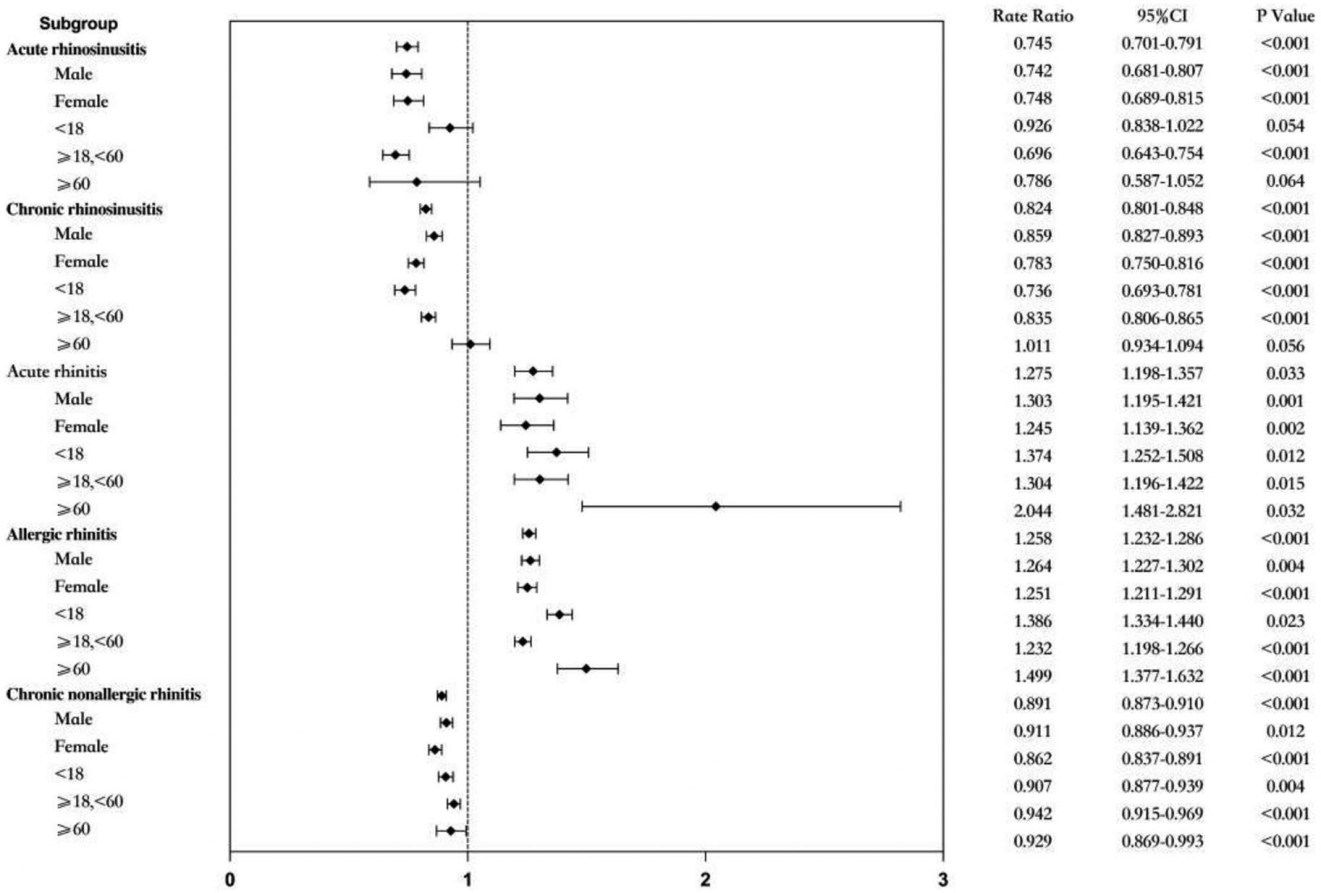
Effects of NPIs on different diseases and populations.

The incidence of chronic nonallergic rhinitis during the COVID-19 pandemic prevention period (2020–2022) significantly reduced compared with the pre-pandemic period (2018–2019) (*p* < .001; Table 1 and Figure 1). The proportion of men in patients with chronic nonallergic rhinitis increased (*p* = .001), whereas the proportion of minors decreased (*p* < .001) (Table 2). NPIs were found to be protective factors for the onset of chronic nonspecific rhinitis (RR = 0.891; 95% CI = 0.873–0.910). Moreover, NPIs had a stronger protective effect in women and minor patients with chronic nonallergic rhinitis compared with men and older adults (Figure 2).

**Table 1.** Comparison of the five diseases in the outpatient department in 2018–2019 with the diseases in 2020–2022 (*n* (%)).

Disease	Number of cases in 2018–2019	Number of cases in 2020–2022
Chronic nonallergic rhinitis	15,441 (9.5%)	16,747 (8.4%)
Allergic rhinitis	12,415 (7.6%)	19,016 (9.6%)
Acute rhinitis	1616 (1.0%)	2508 (1.3%)
Chronic rhinosinusitis	9001 (5.5%)	9029 (4.5%)
Acute rhinosinusitis	2176 (1.3%)	1973 (1.0%)

**Table 2.** Comparison of gender and age distribution of patients with five types of nasal disorders (*n* (%)).

	2018–2019	2020–2022	*χ* ^2^	*p*
*Chronic nonallergic rhinitis*				
Gender			11.418	.001
Male	8446 (54.7%)	9474 (56.6%)		
Female	6995 (45.3%)	7273 (43.4%)		
Age			173.572	<.001
<18 years	5947 (38.5%)	5278 (31.5%)		
≥18 to <60 years	8012 (51.9%)	9712 (58.0%)		
≥60 years	1482 (9.6%)	1757 (10.5%)		
*Allergic rhinitis*				
Gender			2.069	.150
Male	6351 (52.6%)	10,161 (53.4%)		
Female	5884 (47.4%)	8855 (46.6%)		
Age			65.052	<.001
<18 years	3983 (32.1%)	5398 (28.4%)		
≥18 to <60 years	7656 (61.7%)	12,134 (63.8%)		
≥60 years	776 (6.3%)	1484 (7.8%)		
*Acute rhinitis*				
Gender			1.124	.289
Male	820 (50.7%)	1315 (52.4%)		
Female	796 (49.3%)	1193 (47.6%)		
Age			22.242	<.001
<18 years	753 (46.6%)	1012 (40.4%)		
≥18 to <60 years	812 (50.2%)	1363 (54.3%)		
≥60 years	51 (3.2%)	133 (5.3%)		
*Chronic rhinosinusitis*				
Gender			15.091	<.001
Male	4782 (53.1%)	5057 (56.0%)		
Female	4219 (46.9%)	3972 (44.0%)		
Age			164.319	<.001
<18 years	2439 (27.1%)	1755 (19.4%)		
≥18 to <60 years	5528 (61.4%)	5940 (65.8%)		
≥60 years	1034 (11.5%)	1334 (14.8%)		
*Acute rhinosinusitis*				
Gender			0.056	.813
Male	1107 (50.9%)	1011 (51.2%)		
Female	1069 (49.1%)	962 (48.8%)		
Age			1.312	.519
<18 years	805 (37.0%)	729 (36.9%)		
≥18 to <60 years	1281 (58.9%)	1148 (58.2%)		
≥60 years	90 (4.1%)	96 (4.9%)		

The incidence of allergic rhinitis during the COVID-19 pandemic prevention period (2020–2022) significantly increased compared with the pre-pandemic period (2018–2019) (*p* < .001; Table 1 and Figure 1). An increase in the proportion of male patients was observed, but this indicated no statistically significant difference (*p* = .15). In contrast, the proportion of minors decreased (*p* < .001) (Table 2). NPIs were identified as a risk factor for allergic rhinitis (RR = 1.259; 95% CI = 1.232–1.287), and the male and elderly populations were more susceptible to the effects of these measures, leading to an increase in the incidence rate (Figure 2).

The incidence of acute rhinitis significantly increased during the COVID-19 pandemic prevention period (2020–2022) compared with the pre-pandemic period (2018–2019) (*p* < .001; Table 1 and Figure 1). An increase in the proportion of male patients was observed, but this indicated no statistically significant difference (*p* = .289), whereas the proportion of minors decreased (*p* < .001) (Table 2). NPIs were identified as a risk factor for acute rhinitis (RR = 1.276; 95% CI = 1.199–1.357). The analysis indicated that NPIs substantially affected the incidence of acute rhinitis in men and elderly individuals (Figure 2).

The incidence of chronic sinusitis significantly decreased during the COVID-19 pandemic prevention period (2020–2022) compared with the pre-pandemic period (2018–2019) (*p* < .001; Table 1 and Figure 1). The study demonstrated an increase in the proportion of male patients (*p* < .001), while a decrease in the proportion of minors (*p* < .001) (Table 2). NPIs were identified as a protective factor for chronic sinusitis (RR = 0.825; 95% CI = 0.801–0.848), and the RR for elderly people was not statistically significant in patients with chronic sinusitis. Females and minor patients were more likely to benefit from NPIs (Figure 2).

A significant decrease (*p* < .001) in the incidence of acute sinusitis during the COVID-19 pandemic prevention period (2020–2022) was observed compared with the pre-pandemic period (2018–2019) (Table 1 and Figure 1). No statistically significant difference was observed in the age distribution (*p* = .519) or sex proportion (*p* = .813) among patients. NPIs were protective factors for acute sinusitis (RR = 0.745; 95% CI = 0.701–0.792). In patients with acute sinusitis, the RR among elderly and minor patients was not statistically significantly different (Figure 2).

**Figure 3.**
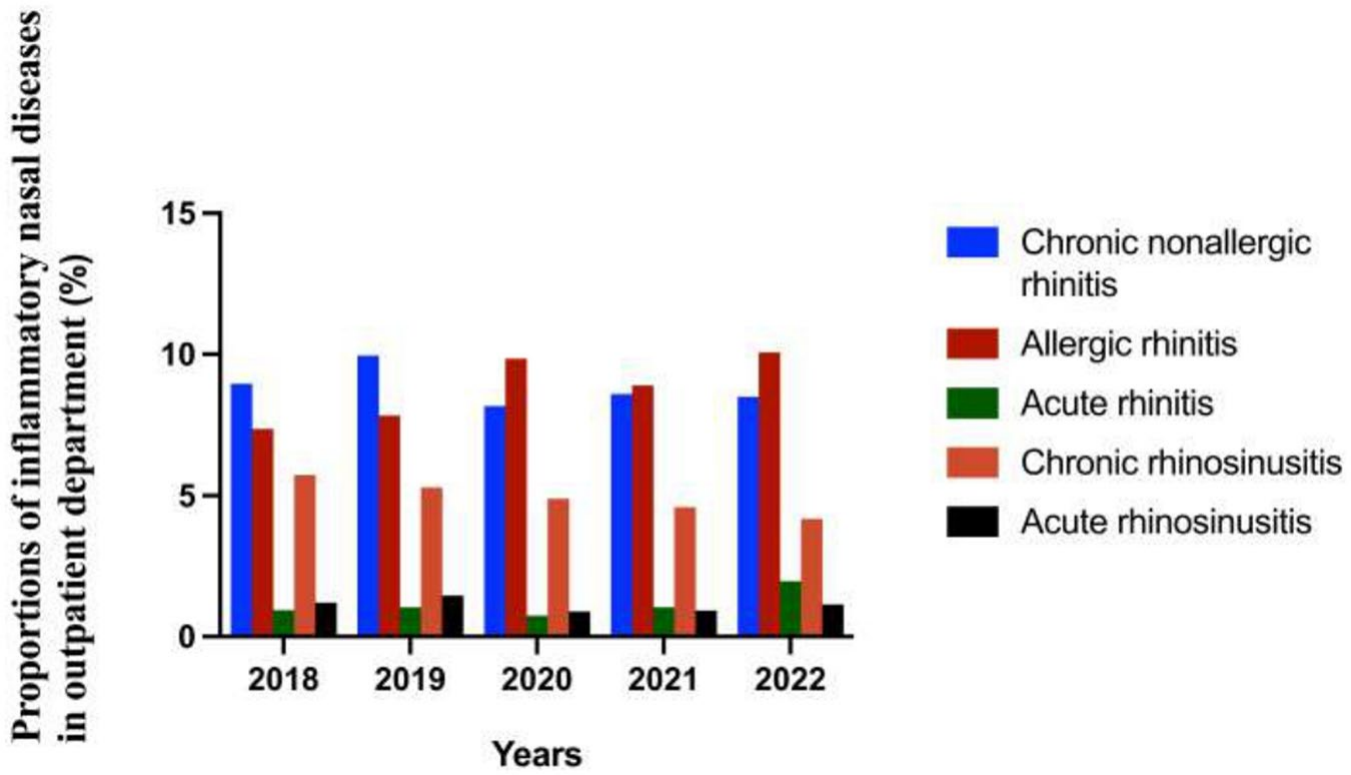
Annual incidence rate of inflammatory nasal diseases in 2018–2022.

**Figure 4.**
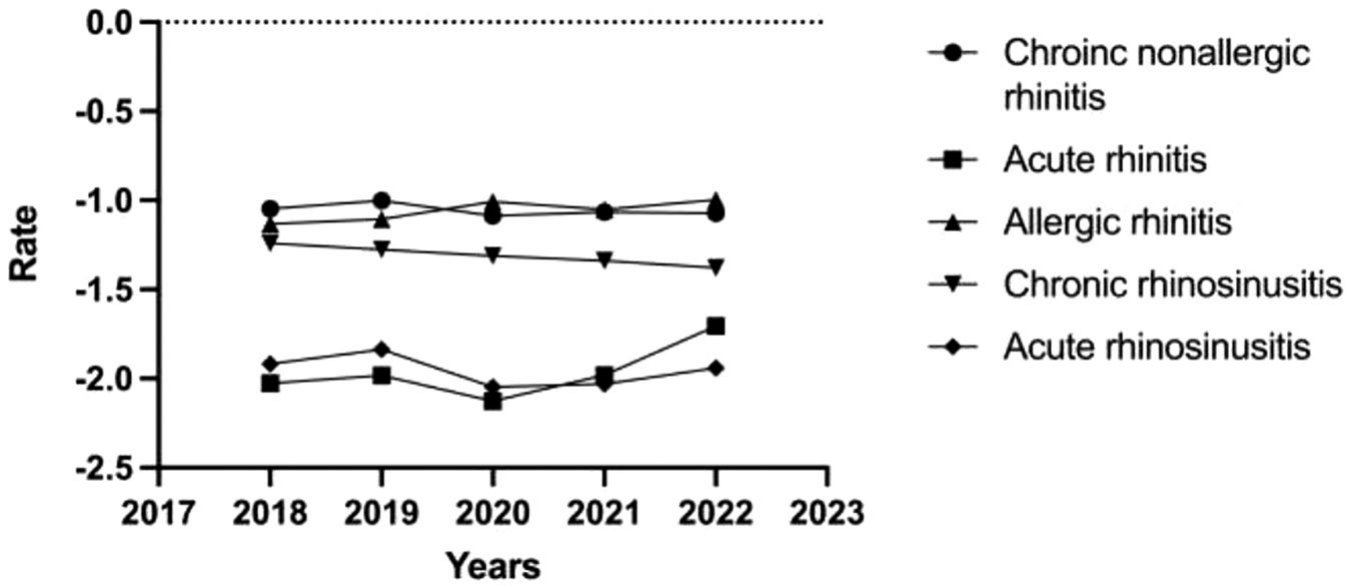
Semi-log line graph showing incidence rates of inflammatory nasal diseases.

We observed that the incidence of the four diseases, except allergic rhinitis, decreased from 2019 to 2020. The incidence of chronic rhinosinusitis decreased gradually from 2018 to 2022. However, the incidence of acute rhinitis increased significantly compared with the other four diseases from 2020 to 2022. Chronic nonallergic rhinitis had the highest incidence rate before the COVID-19 pandemic (2018–2019). However, the incidence rate of allergic rhinitis was the highest during the pandemic prevention period (2020–2022) (see Figures 3 and 4).

## Discussion

The NPIs implemented by the government, such as wearing masks in public, paying attention to hand hygiene, and reducing gatherings, have not only changed people’s lifestyles but also affected their physical and mental health during the pandemic prevention period, thus altering the patterns of clinical disease prevalence [10,11]. Notably, long-term mask-wearing has altered the physical environment of the nasal cavity and sinuses, affecting people’s nasal comfort [5,7].

This study collected data on five common inflammatory diseases of the nasal cavity and sinuses from the Department of Otorhinolaryngology of the Hangzhou Normal University Affiliated Hospital from 2018 to 2022, including chronic nonallergic rhinitis, allergic rhinitis, acute rhinitis, chronic sinusitis and acute sinusitis.

The incidence of allergic rhinitis significantly increased during the pandemic prevention period compared with the pre-pandemic period and ranked first among the five nasal inflammatory diseases, surpassing chronic nonspecific rhinitis. Allergic rhinitis had the highest incidence before the COVID-19 outbreak. NPIs were identified as a risk factor for allergic rhinitis. In 2019–2020, due to strict COVID-19 control measures, the number of patients visiting hospitals decreased sharply and the hospital visits were mainly for emergency cases. Allergic rhinitis had an increased incidence rate among the five nasal inflammatory diseases. This may indicate that allergic rhinitis significantly impacts people’s daily lives and that people are still willing to undertake the risk of infection to receive treatment in hospitals during the pandemic. Jin et al. [10] reported that the percentage of allergic rhinitis cases in 2020 (12.2%) was higher than the average data of the same period in the past three years (7.6%). Furthermore, other studies have also reported similar results [11,12]. This might be the result of the combined effect of environmental, behavioural, immune and psychological factors. Long-term home stay leads to a significant increase in the exposure time and concentration of indoor allergens such as dust mites and pet dander. At the same time, the frequent use of chemical irritants such as disinfectants and cleaning agents directly damages the nasal mucosa and triggers non-IgE-mediated inflammatory responses. Although masks can block some allergens, long-term wearing may aggravate symptoms by changing the nasal mucosa microenvironment (such as increased humidity, CO_2_ retention) or material contact allergies [12,13]. Furthermore, although the psychological factors caused by NPIs such as wearing masks [10] do not directly lead to allergic diseases, the resulting neuroendocrine and immune dysfunction may increase the risk of susceptible individuals developing diseases [14,15]. Under the threat of the epidemic and strict prevention and control measures, people’s psychological and social stress has increased [16]. Stress may intensify mast cell degranulation and Th2-type inflammatory responses by activating the HPA axis and the release of neuropeptides (such as substance P). The reduction of outdoor activities and insufficient exposure to microorganisms further weaken immune tolerance [17]. Additionally, the male and elderly populations have lower compliance and standardization in following NPIs compared with the female and younger populations, making the two earlier groups vulnerable to contact with allergens in the air and environment. Hence, the incidence of allergic rhinitis is higher in male and elderly patients [18] because they are more prone to the impact of NPIs. However, some studies showed that during the blockade period, due to people staying indoors and reducing outdoor activities, exposure to air pollution and allergens decreased, which might lower the incidence of allergic rhinitis [19,20]. Wearing masks may also alleviate the symptoms of allergic rhinitis [8]. However, the data collected in this study were limited to the population in the surrounding area of Hangzhou City, and it was difficult to generalize the observed relationships in this study. More multicentre data may be needed to further explore the impact of NPIs during the pandemic prevention period on the incidence rate of allergic rhinitis.

The incidence of acute rhinitis increased significantly during the COVID-19 prevention period compared with the pre-pandemic period. Furthermore, the incidence of acute rhinitis increased from 2020 to 2022, which was significantly higher than the incidence of the other four diseases. NPIs were identified as a risk factor for the incidence of acute rhinitis. Studies have shown that wearing masks may irritate the nasal mucosa, as some masks may release polypropylene components into the respiratory airflow; polypropylene fibres can be found in the nasal lavage fluid of mask wearers. This suggests that polypropylene may accumulate on the surface of the nasal mucosa, which is prone to causing acute inflammation of the nasal mucosa [4]. Scarano et al. suggested that continuous use of surgical and N95 masks for 1 h can increase the skin temperature of the lower face under the mask. Upon removal of the mask, the skin temperature of the face rapidly decreases within 1 min. The outpatient visit rate for acute rhinitis in this study indicated an increasing trend since 2021. We believe that prolonged mask-wearing may weaken the ability of the nasal mucosa to warm the respiratory airflow, as the nasal mucosa is exposed to a warm and humid environment for a long time. Further, frequent removal and donning of masks can cause abrupt changes in air temperature and humidity around the nasal mucosa, which can make the nasal mucosa sensitive to external air changes and trigger relevant neuroreflexes [21], leading to functional disorders and susceptibility to viruses causing acute rhinitis. Furthermore, several experimental studies have demonstrated that masks create a warm and humid environment that can promote the accumulation of pathogens (bacteria, fungi and viruses) both inside and outside the masks [22–24], leading to clinically relevant fungal, bacterial or viral infections. Champredon et al. reported that wearing masks increased human rhinovirus (HRV) infections [25]. HRV is the most common pathogen that causes acute infection of the nasal mucosa. Because masks may have limited effectiveness in preventing droplet and aerosol transport of HRV [26], and HRV can survive longer than enveloped viruses and easily adhere to the surface of masks, repeated use of masks can easily lead to HRV infection [27]. A number of people are becoming careless about wearing masks with the expansion in the COVID-19 control period in China, often resulting in situations where masks are reused or not removed when sneezing. This secondary pollution of masks may also lead to HRV infection and the symptoms of acute rhinitis. Therefore, we believe that prolonged and improper use of masks were the main reasons for the significant increase in the incidence of acute rhinitis during 2021 and 2022.

The incidence of chronic nonallergic rhinitis, chronic sinusitis and acute sinusitis during the pandemic prevention period significantly decreased compared with the pre-pandemic period. NPIs were identified as protective factors for these three diseases. Microbial infection is the most common cause of sinusitis, which can lead to upper respiratory tract symptoms such as nasal symptoms (nasal secretions, congestion and coughing) [28]. Acute sinusitis is a sinus mucosal infection that occurs concurrently with upper respiratory tract infection. It is most commonly caused by viral or bacterial infections. The pathogenesis of chronic rhinosinusitis is more complex. The traditional view holds that infection, hypersensitivity and anatomical abnormalities of the nasal cavity and sinuses are the three major pathogenic factors. These factors often intersect, but environmental factors, genetic factors, osteitis, gastroesophageal reflux, respiratory cilia system diseases and systemic immunodeficiency can also be contributing factors. The main characteristic of chronic nonallergic rhinitis is nasal mucosal swelling, increased secretions, a course lasting several months or recurrent episodes, and a protracted and refractory course, often without an apparent pathogenic microbial infection. Masks can filter out bacteria of a certain diameter in the environment [29] and can also reduce the amount of bacteria transmitted from mask wearers to others not wearing masks [30]. The results of a cross-sectional study showed that wearing masks could reduce facial contact behaviour, especially touching the eyes, nose and mouth [31], significantly reducing the chance of deep nasal mucosa contact with pathogens and the exposure time of nasal mucosa to pathogens. Behaviours such as wearing masks, maintaining hand hygiene and reducing social activities have reduced the transmission of pathogens between people to a certain extent during the pandemic prevention period, reducing the possibility of sinus mucosal infection, and led to a decrease in the incidence of acute and chronic sinusitis. Moreover, the onset of chronic nonallergic rhinitis, acute sinusitis and chronic sinusitis is relatively slow, and may have a relatively small impact on people’s daily lives. Patients’ tolerance of their symptoms is relatively high, and people tend to avoid seeking medical treatment or opt for online consultations or self-medication under the risk of pandemic infection.

In the diagnosed population of chronic non-allergic rhinitis and chronic sinusitis, the proportion of male patients is increased and significantly significant. Although the proportion of male patients with allergic rhinitis, acute rhinitis and acute sinusitis also increased, it indicated no statistically significant difference. Traditional sex norms in society may be related to the standardization of protective measures. In traditional societies, men are expected to display strength rather than vulnerability, which is why men are more likely to engage in risky behaviours compared with women [32]. Studies related to wearing masks during previous pandemics showed that men were less likely to wear masks than women during influenza A virus (H1N1) [33,34] and severe acute respiratory syndrome (SARS) [35] pandemics. Similarly, studies focusing on wearing masks during the COVID-19 pandemic showed that those who strongly identify with the idea that men should be strong are more likely to have negative emotions toward wearing masks [36]. Compliance with wearing masks may be lower among men than women, and men may be more likely to ignore the standardization of wearing masks. These studies suggested that men were less likely than women to comply with NPIs, which might explain the observed phenomenon of a higher proportion of male patients.

Among patients with inflammatory diseases in the nasal region, the proportion of minors decreased during the pandemic prevention period compared with the pre-pandemic period, with statistically significant differences in the age of patients with the other four diseases except for acute sinusitis. A study published in the *Journal of Medical Virology* found that during the pandemic period, the diagnosis rate of infectious diseases in children decreased significantly and was more pronounced than in adults [37], which was consistent with our findings. Children are believed to have lower immunity than adults and are more susceptible to pathogenic infections. Hence, families and society are paying more attention to standardizing children’s mask-wearing and hand hygiene. Emphasizing the measures that can reduce pathogen transmission may be more beneficial for children’s health during the pandemic prevention period. Furthermore, a previous study has identified respiratory syncytial virus (RSV) as the main pathogen for children’s respiratory infections [38]. Leung et al. found that using masks could reduce the transmission of RSV [26]. The COVID-19 pandemic resulted in the suspension of teaching courses in most schools, significantly reducing the social activities of minors and hence reducing the transmission of susceptible viruses among minors. These reasons may explain the phenomenon of the decrease in the proportion of minor patients during the pandemic prevention period.

## Conclusions

**Figure 5.**
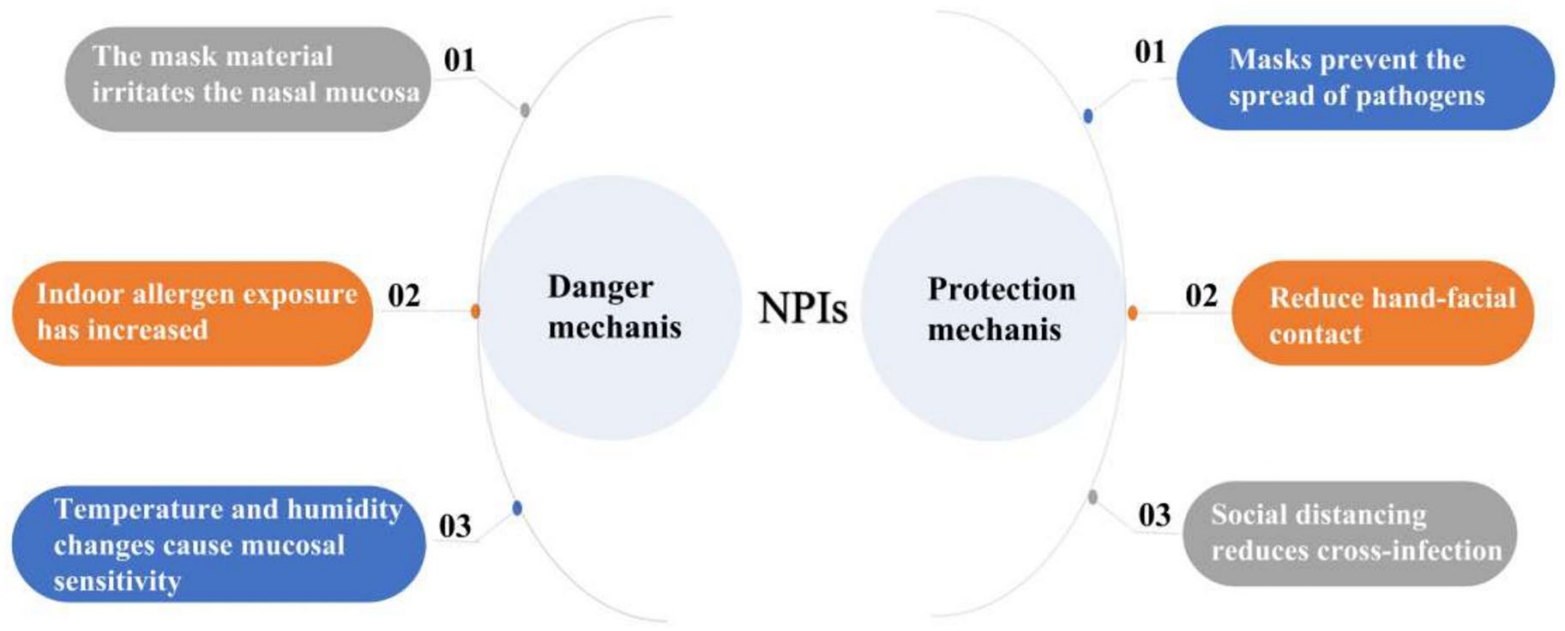
The double impact of NPIs on nasal inflammatory diseases.

The novel coronavirus can lead to diseases, and NPIs implemented by the government can also affect people’s lives and behaviour patterns, resulting in changes in the epidemiology of clinical diseases. The data supporting the use of masks continue to emerge. For example, a study investigated the different sources of variability in COVID-19-induced per capita mortality rates in more than 200 countries and reported a negative correlation between the duration of public mask-wearing and mortality rates [39]. Studies also indicated that combining NPIs such as lockdowns and mask-wearing could reduce the COVID-19 mortality rate and alleviate the demand for medical care [40]. Although restrictions on wearing masks in public places have been relaxed in many countries, NPIs such as mask-wearing and lockdowns may reappear in future respiratory disease pandemics. Furthermore, in certain professions such as doctors and nurses, wearing masks and paying attention to hand hygiene may become routine. This study provided some insights to better understand changes in nasal diseases under NPIs, the general mechanism of action is shown in Figure 5. It may help people recognize the dual nature of NPIs and improve the understanding of the necessity of patients with susceptible nasal inflammatory diseases to wear masks in daily life and reduce outdoor activities to reduce the incidence of nasal mucosa diseases.

## Supplementary Material

（clean version）Impact of nonpharmaceutical interventions on inflammatory diseases of the nose.docx

## Data Availability

The raw data supporting the conclusions of this article will be made available by Lili Dai at daill_333@163.com, without undue reservation.

## References

[1] Zhou P, Yang X-L, Wang X-G, et al. A pneumonia outbreak associated with a new coronavirus of probable bat origin. Nature. 2020;579(7798):270–273. doi:10.1038/s41586-020-2012-7. 32015507 PMC7095418

[2] Xu T-L, Ao M-Y, Zhou X, et al. China’s practice to prevent and control COVID-19 in the context of large population movement. Infect Dis Poverty. 2020;9(1):115. doi:10.1186/s40249-020-00716-0. 32814591 PMC7435224

[3] Wang Q, Huang L. China’s “dynamic clearing” epidemic prevention policy: achievements, challenges, and prospects. Front Public Health. 2022;10:978944. doi:10.3389/fpubh.2022.978944. 36187631 PMC9516291

[4] Klimek L, Huppertz T, Alali A, et al. A new form of irritant rhinitis to filtering facepiece particle (FFP) masks (FFP2/N95/KN95 respirators) during COVID-19 pandemic. World Allergy Organ J. 2020;13(10):100474. doi:10.1016/j.waojou.2020.100474. 33042359 PMC7538121

[5] Primov-Fever A, Amir O, Roziner I, et al. How face masks influence the sinonasal quality of life during the COVID-19 pandemic. Eur Arch Otorhinolaryngol. 2021;278(12):4805–4811. doi:10.1007/s00405-021-06752-2. 33772607 PMC7998091

[6] Deng W, Sun Y, Yao X, et al. Masks for COVID‐19. Adv Sci. 2021;9:2102189. doi:10.1002/advs.202102189. PMC8787406 34825783

[7] Scarano A, Inchingolo F, Lorusso F. Facial skin temperature and discomfort when wearing protective face masks: thermal infrared imaging evaluation and hands moving the mask. Int J Environ Res Public Health. 2020;17(13):4624. doi:10.3390/ijerph17134624. 32605056 PMC7369838

[8] Dror AA, Eisenbach N, Marshak T, et al. Reduction of allergic rhinitis symptoms with face mask usage during the COVID-19 pandemic. J Allergy Clin Immunol Pract. 2020;8(10):3590–3593. doi:10.1016/j.jaip.2020.08.035. 32889221 PMC7467086

[9] Olsen SJ, Winn AK, Budd AP, et al. Changes in influenza and other respiratory virus activity during the COVID-19 pandemic—United States, 2020–2021. MMWR Morb Mortal Wkly Rep. 2021;70(29):1013–1019. doi:10.15585/mmwr.mm7029a1. 34292924 PMC8297694

[10] Jin L, Fan K, Tan S, et al. Analysis of the characteristics of outpatient and emergency diseases in the department of otolaryngology during the “COVID-19” pandemic. Sci Prog. 2021;104(3):368504211036319. doi:10.1177/00368504211036319. 34323155 PMC10358545

[11] Koshevarova VA, Westenhaver ZK, Schmitz-Brown M, et al. Blepharoconjunctivitis and otolaryngological disease trends in the context of mask wearing during the COVID-19 pandemic. Clin Pract. 2022;12(4):619–627. doi:10.3390/clinpract12040065. 36005068 PMC9406373

[12] Gelardi M, Trecca EMC, Fortunato F, et al. COVID-19: when dust mites and lockdown create the perfect storm. Laryngosc Investig Otolaryngol. 2020;5(5):788–790. doi:10.1002/lio2.439. PMC7436479 32838034

[13] Kushnir-Sukhov NM. A novel link between early life allergen exposure and neuroimmune development in children. J Clin Exp Immunol. 2020;5:188–195. doi:10.33140/jcei.05.04.06. 33179020 PMC7654965

[14] Timonen M, Jokelainen J, Silvennoinen-Kassinen S, et al. Association between skin test diagnosed atopy and professionally diagnosed depression: a Northern Finland 1966 Birth Cohort study. Biol Psychiatry. 2002;52:349–355. doi:10.1016/s0006-3223(01)01364-6. 12208642

[15] Postolache TT, Komarow H, Tonelli LH. Allergy: a risk factor for suicide? Curr Treat Options Neurol. 2008;10(5):363–376. doi:10.1007/s11940-008-0039-4. 18782509 PMC2592251

[16] Chamaa F, Bahmad HF, Darwish B, et al. PTSD in the COVID-19 era. Curr Neuropharmacol. 2021;19(12):2164–2179. doi:10.2174/1570159X19666210113152954. 33441072 PMC9185760

[17] Joachim RA, Cifuentes LB, Sagach V, et al. Stress induces substance P in vagal sensory neurons innervating the mouse airways. Clin Exp Allergy. 2006;36(8):1001–1010. doi:10.1111/j.1365-2222.2006.02533.x. 16911356

[18] Tang H, Wang J, Zhang Y, et al. Knowledge and behaviour of community residents’ face mask-wearing during the COVID-19 pandemic: a cross-sectional study in Shanghai, China. BMJ Open. 2022;12(2):e052497. doi:10.1136/bmjopen-2021-052497. PMC8844952 35149563

[19] Dayal AK, Sinha V. Trend of allergic rhinitis post COVID-19 pandemic: a retrospective observational study. Indian J Otolaryngol Head Neck Surg. 2022;74(1):50–52. doi:10.1007/s12070-020-02223-y. 33102190 PMC7575215

[20] Choi HG, Kim SY, Joo Y-H, et al. Incidence of asthma, atopic dermatitis, and allergic rhinitis in Korean adults before and during the COVID-19 pandemic using data from the Korea National Health and Nutrition Examination Survey. Int J Environ Res Public Health. 2022;19(21):14274. doi:10.3390/ijerph192114274. 36361154 PMC9658105

[21] Canning BJ. Neurology of allergic inflammation and rhinitis. Curr Allergy Asthma Rep. 2002;2:210–215. doi:10.1007/s11882-002-0021-2. 11918862

[22] Chughtai AA, Stelzer-Braid S, Rawlinson W, et al. Contamination by respiratory viruses on outer surface of medical masks used by hospital healthcare workers. BMC Infect Dis. 2019;19(1):491. doi:10.1186/s12879-019-4109-x. 31159777 PMC6547584

[23] Luksamijarulkul P, Aiempradit N, Vatanasomboon P. Microbial contamination on used surgical masks among hospital personnel and microbial air quality in their working wards: a hospital in Bangkok. Oman Med J. 2014;29(5):346–350. doi:10.5001/omj.2014.92. 25337311 PMC4202234

[24] Zhiqing L, Yongyun C, Wenxiang C, et al. Surgical masks as source of bacterial contamination during operative procedures. J Orthop Translat. 2018;14:57–62. doi:10.1016/j.jot.2018.06.002. 30035033 PMC6037910

[25] Champredon D, Bancej C, Lee L, et al. Implications of the unexpected persistence of human rhinovirus/enterovirus during the COVID-19 pandemic in Canada. Influenza Other Respir Viruses. 2022;16(2):190–192. doi:10.1111/irv.12930. 34747155 PMC8652650

[26] Leung NHL, Chu DKW, Shiu EYC, et al. Respiratory virus shedding in exhaled breath and efficacy of face masks. Nat Med. 2020;26(5):676–680. doi:10.1038/s41591-020-0843-2. 32371934 PMC8238571

[27] Jia R, Lu L, Li S, et al. Human rhinoviruses prevailed among children in the setting of wearing face masks in Shanghai, 2020. BMC Infect Dis. 2022;22(1):253. doi:10.1186/s12879-022-07225-5. 35287614 PMC8919361

[28] Meltzer EO, Hamilos DL, Hadley JA, et al. Rhinosinusitis: establishing definitions for clinical research and patient care. J Allergy Clin Immunol. 2004;114(6 Suppl.):155–212. doi:10.1016/j.jaci.2004.09.029. 15577865 PMC7119142

[29] Davies A, Thompson K-A, Giri K, et al. Testing the efficacy of homemade masks: would they protect in an influenza pandemic? Disaster Med Public Health Prep. 2013;7(4):413–418. doi:10.1017/dmp.2013.43. 24229526 PMC7108646

[30] Greene VW, Vesley D. Method for evaluating effectiveness of surgical masks. J Bacteriol. 1962;83:663–667. doi:10.1128/jb.83.3.663-667.1962. 13901536 PMC279325

[31] Chen Y-J, Qin G, Chen J, et al. Comparison of face-touching behaviors before and during the coronavirus disease 2019 pandemic. JAMA Netw Open. 2020;3(7):e2016924. doi:10.1001/jamanetworkopen.2020.16924. 32725247 PMC12124488

[32] Mahalik JR, Burns SM, Syzdek M. Masculinity and perceived normative health behaviors as predictors of men’s health behaviors. Soc Sci Med. 2007;64(11):2201–2209. doi:10.1016/j.socscimed.2007.02.035. 17383784

[33] Condon BJ, Sinha T. Who is that masked person: the use of face masks on Mexico City public transportation during the influenza A (H1N1) outbreak. Health Policy. 2010;95(1):50–56. doi:10.1016/j.healthpol.2009.11.009. 19962777

[34] Lau JTF, Griffiths S, Choi K-C, et al. Prevalence of preventive behaviors and associated factors during early phase of the H1N1 influenza epidemic. Am J Infect Control. 2010;38(5):374–380. doi:10.1016/j.ajic.2010.03.002. 20569849 PMC7132693

[35] Tang CS, Wong C. Factors influencing the wearing of facemasks to prevent the severe acute respiratory syndrome among adult Chinese in Hong Kong. Prev Med. 2004;39(6):1187–1193. doi:10.1016/j.ypmed.2004.04.032. 15539054 PMC7133369

[36] Palmer CL, Peterson RD. Toxic mask-ulinity: the link between masculine toughness and affective reactions to mask wearing in the COVID-19 era. Polit Gend. 2020;16(4):1044–1051. doi:10.1017/S1743923X20000422.

[37] Tanislav C, Kostev K. Fewer non‐COVID‐19 respiratory tract infections and gastrointestinal infections during the COVID‐19 pandemic. J Med Virol. 2022;94(1):298–302. doi:10.1002/jmv.27321. 34491581 PMC8661971

[38] Li Z-J, Zhang H-Y, Ren L-L, et al. Etiological and epidemiological features of acute respiratory infections in China. Nat Commun. 2021;12(1):5026. doi:10.1038/s41467-021-25120-6. 34408158 PMC8373954

[39] Leffler CT, Ing E, Lykins JD, et al. Association of country-wide coronavirus mortality with demographics, testing, lockdowns, and public wearing of masks. Am J Trop Med Hyg. 2020;103(6):2400–2411. doi:10.4269/ajtmh.20-1015. 33124541 PMC7695060

[40] Geng Y, Zhang L. Impact of non-pharmaceutical interventions during COVID-19 pandemic on pertussis, scarlet fever and hand-foot-mouth disease in China. J Infect. 2022;84(2):e13–e15. doi:10.1016/j.jinf.2021.12.023. PMC8694816 34953908

